# Study on the Economic Burden of Neurodevelopmental Diseases on Patients With Genetic Diagnosis

**DOI:** 10.3389/fpubh.2022.887796

**Published:** 2022-05-09

**Authors:** Donghua Xie, Ruoyu Duan, Chen Li, Zhiqun Xie, Aihua Wang, Lili Xiong, Jianhui Wei, Hui Xi, Junqu Fang, Huifang Yan, Junyu Wang, Yu Zhang, Xiao Mao, Jingmin Wang, Hua Wang

**Affiliations:** ^1^Department of Information Management, Maternal and Child Health Hospital of Hunan Province, Changsha, China; ^2^Department of Pediatrics, Peking University First Hospital, Beijing, China; ^3^Department of Neurology, National Center for Children's Health, Beijing Children's Hospital, Capital Medical University, Beijing, China; ^4^Children Health Hospital of Shenzhen City, Shenzhen, China; ^5^Xiangya School of Public Health, Central South University, Changsha, China; ^6^NHC Key Laboratory of Birth Defect for Research and Prevention (Maternal and Child Health Hospital of Hunan Province), Changsha, China; ^7^Beijing Key Laboratory of Molecular Diagnosis and Study on Pediatric Genetic Diseases, Beijing, China; ^8^Key Laboratory for Neuroscience, Ministry of Education/National Health and Family Planning Commission, Peking University, Beijing, China

**Keywords:** genetic diagnosis, neurodevelopmental disease, cost, insurance, economic burden

## Abstract

**Objective:**

To study the burden of neurodevelopmental diseases (NDDs) via cost-of-illness analysis of Chinese patients with genetic diagnosis.

**Methods:**

We recruited NDD patients (0–18 years old) with genetic diagnosis (GD) from September 1, 2020 to January 30, 2021. We gathered basic information on the details of diagnosis, as well as the direct medical cost, direct non-healthcare cost and indirect cost before and after receiving GD. We corrected the cost for time biases by calculating the cost per day for each patient.

**Results:**

For the 502 patients with NDDs, the mean age was 4.08 ± 3.47. The household income was 0.6 (0.4, 1.0) 10,000 CNY per-month on average. The direct medical cost, direct non-healthcare cost and indirect cost were 12.27 (7.36, 22.23) 10,000 CNY, 1.45 (0.73, 2.69)10,000 CNY and 14.14(4.80, 28.25) 10,000 CNY per patient, respectively. Every patient received 1.20 (0.34, 3.60) 10,000 CNY on average (15.91%) from insurance. The daily total cost after receiving GD were ~62.48% lower than those before GD (191.59 CNY vs. 71.45 CNY). The descend range of lab cost (95.77%, *P* < 0.05) was the largest, followed by drugs (91.39%, *P* < 0.05), hospitalization (90.85%, *P* < 0.05), and consultation (57.41%, *P* < 0.05). The cost of rehabilitation kept slightly increasing but there were no significant differences (*P* > 0.05). The daily direct medical cost of each patient fell by 75.26% (*P* < 0.05) from 311.79 CNY to 77.14 CNY when the diagnostic age was younger than 1, and declined by 49.30% (*P* < 0.05) and 8.97% (*P* > 0.05) when the diagnostic age was 1–3 and older than 3, respectively.

**Conclusions:**

Early genetic diagnosis is crucial for to reducing the burden of disease because of the amount of money spent was lower when they are diagnosed at younger age. Patients with NDDs can incur a heavy economic burden, especially in rehabilitation cost and indirect cost, because the insurance coverage for patients is low, so it is urgent for governments to pay more attention to these issues.

## Introduction

Neurodevelopmental diseases (NDDs) involve the central nervous system (CNS). NDDs are mostly found in children (1) and seriously influence their quality of life. To date, NDDs affect more than 3% of children (2). NDDs not only cause difficulties for children's physical and psychological health, but also serious socioeconomic hardship (3). NDDs have clinical and genetic heterogeneity. Early diagnosis, especially genetic diagnosis (GD), has great significance to reduce the burden of disease (4). Therefore, an accurate and quick diagnosis is urgent, especially for treatable NDDs. Based on the improvement of genetic testing techniques, an increasing number of NDD patients have been diagnosed by chromosomal microarray analysis (CMA), gene panels, whole exon sequencing (WES), whole genome sequencing (WGS), and genome copy number variation (CNV). Questions on affordability and cost impact for society have also been raised (5–7). Many NDDs can be diagnosed with several different testing methods, and few NDDs involve therapeutic techniques (4). Therefore, these diagnostic methods in the clinic still raise questions on affordability and cost impact for society as a whole (5, 8). Many patients and some doctors in primary hospitals believe that even if the causal gene is identified (which is costly), an exact therapeutic method cannot be determined. Therefore, to some extent, the diagnostic trajectory may be prolonged, or many children may go undiagnosed (4).

GD methods have high cost-effectiveness in NDDs for several studies (4, 9). Vrijenhoek et al. showed that the post-WES cost were 80% lower per patient than the pre-WES cost (5). A study from Australia on NDD disease burden illustrated that the use of WES in infants resulted in a gain of 7.39 quality-adjusted life-years (QALYs) and an incremental cost-effectiveness ratio (ICER) of AU $31,144.35(9). The diagnostic rate of a cohort of 50 patients with early-onset epileptic encephalopathy was 42% in Ireland (10). The average cost of WES was €25,00 and could save €64 837 if WES were performed as a first-line investigation in the entire cohort.

China is a developing country with 1.4 billion people. To date, there has been no research about the benefit of GD in NDDs. Additionally, existing studies do not consider the main influencing factors of cost-effectiveness such as sex, county, age, income, and health insurance. In addition, they do not take into account the direct non-healthcare cost of visiting hospitals, including transportation, accommodations, catering and indirect cost of, productivity losses due to caring which also bring heavy economic burden. We aimed to analyze the economic impact, including the direct medical cost, direct non-healthcare cost and indirect cost of GD, based on a large sample of patients with NDDs. We also compared the different benefits between subgroups according to their characteristics. This study provides scientific proof that the government should develop policies about promoting genetic testing for NDD.

## Methods

### Study Design

Our research was retrospective economics survey about positive case. We used pre-cost and post-cost comparisons for confirmed cases.

### Patients

We recruited children aged 0 to 18 from September 1, 2020 to January 30, 2021 at the Children's Health Hospital of Hunan Province, the Children's Health Hospital of Shenzhen City, and Peking University First Hospital through the outpatient departments and websites of the three hospitals. The inclusion criterion was as follows: patients diagnosed as NDDs with specific genetic mutations. Exclusion criteria were: Parents forgot the total cost completely and could not find out the credentials of the cost. Parents were unwilling to cooperate. We recruited a total of 540 patients with NDDs who met the criteria, of whom we eliminated 38 due to invalid questionnaires with too many logical errors. Finally, we included 502 questionnaires (effective rate: 92.96%). This study was approved by the Ethics Committee of Maternal and Child Care at the Hospital of Hunan Province. All patients' parents signed letters of informed consent.

### Patient and Public Involvement

Firstly, we asked for several parents with NDD patients about the structure of cost and invited them as assistants of this research. Secondly, all patients had to upload their clinical and genetic diagnosis certifications. Geneticist pediatric neurologists reviewed them carefully and gave patients and their families a free consultation.

### Data Collection and Quality Control

We took all hospital-related cost for the patients' NDDs into account. We collected the details on cost through an electronic questionnaire survey, which included three parts. The first part contained basic information on the children such as birth data, residence, sex, as well as the parents' age, education, occupation, and income. The second part covered the details of diagnosis, including clinical diagnosis, genetic diagnosis, diagnostic age, diagnostic hospital, diagnostic methods, and diagnostic cost. The third part entailed the cost of outpatient visits, inpatient visits, rehabilitation, catering, accommodations, transportation, and productivity losses due to caring before and after the GD separately. We classified the cost of outpatient visits into 3 categories (registration, other laboratory tests, drugs). We excluded cost not related to the NDDs.

Before completing the questionnaire, we designed detailed instructions and an electronic questionnaire survey with logical relationships to ensure quality. Most of the parents could provide a list of major cost. Additionally, our pediatrician checked the questionnaire once the parents finished filling it out. If there were mistakes or doubts, we contacted the parents and taught them how to make corrections or asked them to fill it out again.

### Statistical Analysis

We calculated direct medical cost (including outpatient, inpatient, and rehabilitation cost) and direct non-healthcare cost (including catering, accommodations, transportation) and indirect economic burdens (productivity losses due to care delays). To assess the impact of GD on post-test health care activities, we assessed the cost of total and different age groups before and after receiving the GD. According to the children's developmental characteristics, we classified the ages into the following groups: <3 years old, 3–6 years old, and ≥6 years old. There was a large difference in the time length of patients diagnosed before and after GD, So we corrected for time biases by calculating the cost per day for each patient (8) and compared it between features of different groups, including diagnostic age, sex, county, one-child family, parents' education level, and income. In addition, we calculated the cost and times, distance of diagnostic location, and medical insurance. We used frequency (%)//median and quartile range (QR) for descriptive analysis. We used the Wilcoxon matched-pairs signed-rank test to compare the different impacts of the total, per day, and detailed cost after the GD between groups with different characteristics. Kruskal–Wallis H test was used to compare the different impacts of different age group. We analyzed the data using SPSS 25.0 (SPSS, Chicago, IL) with a significance level of *P* < 0.05.

## Result

### Characteristics of the Population and Basic Information on GD and Medical Insurance

Among the 502 patients with NDDs, the mean age was 4.08 **±** 3.47 years old, 47.41% patients were urban residents, and 55.58% children were from one-child families. The mean age of their parents was (32.75 ± 5.70 and 34.63 ± 5.59), and their education level mostly was senior high school/college (41.83, 44.42%) (Table 1).

**Table 1:** Characteristics of the population (*n* = 502) and basic information on GD and medical insurance.

**Items**	**M (P_**25**_, P_**75**_)/**$\bar{x}$ **±s**	***N* (%)**
**1. Characteristic**		
**Age**		
<3 years	1.50 (0.93, 2.24)	238 (47.41)
≥3 years and <6 years	4.16 (3.57, 4.48)	159 (31.67)
≥6 years	8.50 (7.15, 10.80)	105 (20.92)
**Sex**		
Male		281 (55.98)
Female		221 (44.02)
**County**		
Suburban		305 (60.76)
Urban		197 (39.24)
**One-child family**		
Yes		279 (55.58)
No		223 (44.42)
**Age of mother**	32.75 ± 5.70	
**Age of father**	34.63 ± 5.59	
**Education level of the mother**		
Junior high school or below		132 (26.29)
Senior high school/college		210 (41.83)
Undergraduate or above		160 (31.88)
**Education level of the father**		
Junior high school or below		121 (24.10)
Senior high school/college		223 (44.42)
Undergraduate or above		158 (31.47)
**2. Diagnostic Information**		
Diagnostic age (M)	12 (6, 24)	
Number of outpatient visits	6 (4, 12)	
Number of inpatient visits	2 (1, 4)	
Number of genetic tests	0.89 (0.60, 1.20)	
**The location of the diagnostic hospital**		
Same district		10 (1.99)
Same city		101 (20.12)
Same province		97 (19.32)
Trans-provincial		294 (58.57)
**3. Information on direct medical security**		
**Average monthly household income (10,000 CNY)**	0.60 (0.40, 1.00)	
Cost per patient per month	0.69 (0.33,1.55)	
Total cost per patient	12.27 (7.37,22.22)	
Based on medical insurance	1.20 (0.34, 3.60)	

To be diagnosed by GD, a child needed 6 (4, 12) outpatient visits and 6 (4, 12) inpatient visits on average, and 61.19% children needed trans-provincial care. The monthly household income was 0.60 (0.40, 1.00) 10,000 CNY on average, and the direct medical cost per patient per month was 0.69 (0.33, 1.55) 10,000 CNY. The total direct medical cost was 12.27 (7.37, 22.22) 10,000 CNY per patient. Through various medical insurance and aid policies, one child received only 1.20 (0.34, 3.60) 10,000 CNY on average, which was only 15.91% of the total cost (Table 1).

### Total Direct Medical Cost, Direct Non-healthcare Cost and Indirect Medical Cost on Average

The total cost of direct medical care was 12.27 (7.37, 22.22) 10,000 CNY per patient on average. Among these cost, the average cost before GD was 6.45 (3.55, 11.72) 10,000 CNY (51.80%), and after GD, it was 4.19 (1.62, 10.38) 10,000 CNY (48.20%). Among total direct medical cost, cost per category varied considerably, with hospitalization and rehabilitation accounting for the largest portions of the total cost (29.81 and 43.80%, respectively). Compared to the cost before GD, the percentage of outpatient examinations fell from 13.94 to 8.35%, and the rehabilitation rate increased from 35.98 to 52.19% (Table 2).

**Table 2:** The total economic burden on average for NDDs in children (CNY, %) in different age groups.

**Items**	**Group**	**Pre-GD cost (CNY)**	**%**	**Post-cost (CNY)**	**%**	**Total cost (CNY)**	**%**	**Z**	***P***
Diagnosis	<3 years^*^	8,500.00	4.80	-	-	8,500.00	2.49	-	-
	≥3 years and <6 years^*^	9,000.00	3.68	-	-	9,000.00	1.90	-	-
	≥6 years	9000.00	2.47	-	-	9000.00	1.28	-	-
	Sum	8,920.00	10.94	-	-	8,920.00	5.67	-	**-**
Consultation^*^	<3 years	345.00	0.41	240.00	0.36	812.5	0.38	−2.258	0.024
	≥3 years and <6 years	500.00	0.45	400.00	0.53	1,530.00	0.49	−1.892	0.059
	≥6 years	500.00	0.28	400.00	0.22	1,044.00	0.25	−2.390	0.017
	Sum	470.00	1.14	327.00	1.11	1,000.00	1.12	−3.730	<0.001
Lab^*^	<3 years	5,000.00	4.37	2,000.00	2.49	7,900.00	3.46	−6.019	<0.001
	≥3 years and <6 years	8,000.00	5.06	3,240.00	3.38	14,100.00	4.25	−5.030	<0.001
	≥6 years	10,000.00	4.51	4,000.00	2.48	16,000.00	3.53	−3.975	<0.001
	Sum	6,350.00	13.94	2,500.00	8.35	11,225.00	11.24	−8.736	<0.001
Drugs^*^	<3 years	1,500.00	1.63	900.00	1.93	3,000.00	1.77	−1.562	0.118
	≥3 years and <6 years	3,600.00	2.51	3,000.00	3.52	10,000.00	3.00	−0.040	0.968
	≥6 years	5000.00	3.20	2500.00	3.99	12000.00	3.58	−1.535	0.125
	Sum	2,400.00	7.34	1,500.00	9.45	5,795.00	8.36	−1.970	0.049
Hospitalization^*^	<3 years	20,000.00	15.02	5,000.00	10.68	32,000.00	12.93	−5.221	<0.001
	≥3 years and <6 years	15,000.00	9.20	5,000.00	8.47	30,000.00	8.85	−3.068	0.002
	≥6 years	10,000.00	6.44	2,300.00	9.75	24,000.00	8.03	−1.974	0.048
	Sum	16,000.00	30.66	4,550.00	28.90	30,000.00	29.81	−6.001	<0.001
Rehabilitation^*^	<3 years	3,000.00	9.48	4,998.50	11.20	12,970.00	10.31	−1.515	0.13
	≥3 years and <6 years	13,000.00	13.07	18,497.00	19.12	42,000.00	15.98	−0.805	0.421
	≥6 years	22,000.00	13.44	35,000.00	21.87	86,000.00	17.50	−1.713	0.087
	Sum	8,750.00	35.98	12,000.00	52.19	34,500.00	43.80	−2.339	0.019
Total of direct medical expenses^*^	<3 years	50,235.50	35.70	29,020	26.67	99,575.00	31.35	−5.735	<0.001
	≥3 years and <6 years	74,800.00	33.96	55,020	35.02	14,0908.00	34.47	−2.305	0.021
	≥6 years	88,908.00	30.34	6,5932	38.32	22,4120.00	34.18	−0.599	0.549
	Sum	64,525.00	100.00	41,819.50	100.00	122,700.00	100.00	−5.221	<0.001
Direct non-healthcare cost^*^	Transportation	3,000	37.16	2,000	35.07	5,650	36.15	−4.508	<0.001
	Accommodation	2,000	34.09	1,000	34.30	3,500	34.18	−4.554	<0.001
	Catering	1,690	28.75	1,000	30.63	3,300	29.65	−3.651	<0.001
Indirect medicalt^*^	Productivity losses due to caring	33,000	——	68,789.40	——	141,408.50	——	−6.745	<0.001

* *The Kruskal–Wallis H test showed a significant difference in various total costs between different age group*.

The Kruskal–Wallis H test analysis indicated differences in the total direct medical cost of the three age groups (χ^2^ = 56.278, *P* < 0.001). The older the child was, the greater the total direct medical expenses. The average direct medical cost per case was 9.96 (5.86, 149,100) 10,000 CNY for the group younger than 3 years old and 22.41 (9.57, 40.45) 10,000 CNY for the group ≥6 years old.

The total direct non-healthcare cost was 1.45 (0.73, 2.69) 10,000 CNY per patient. The indirect cost (productivity losses) was 14.14(4.80, 28.25) 10,000 CNY per patient. After GD, the share of transportation, accommodations declined by 5.38% (Table 2).

### Comparison of Direct Medical Cost per Day per Patient Before and After GD

The patients survived for 360 (180,720) days before GD and 620 (229,1167) days after GD on average. To make a valid comparison between the cost accrued before and after GD, we subsequently calculated the average per day per patient cost for each category (Tables 3, 4). On average, the daily total cost was 62.48% lower than it was before the GD (191.59 CNY vs. 71.45 CNY). The lab cost (95.77%, *P* < 0.05) was the largest, followed by drugs (91.39%, *P* < 0.05), hospitalization (90.85%, *P* < 0.05), and consultation (57.41%, *P* < 0.05). The cost of rehabilitation increased slightly, but there were no significant differences (*P* > 0.05).

**Table 3:** Direct medical cost per day per patient before GD (CNY).

	**Consultation**	**Lab**	**Drugs**	**Hospitalization**	**Rehabilitation**	**Totally**
**Diagnostic age**						
<1 years old	2.58	33.33	9.09	100.00	7.14	311.79
≥1 years and <3 years	0.94	12.00	5.03	26.38	33.33	131.73
≥3 years	0.30	9.26	3.33	7.55	22.83	87.19
**Sex**						
Male	1.29	19.44	6.67	39.68	30.43	182.78
Female	1.39	17.50	6.67	55.56	14.29	199.00
**County**						
Suburban	1.25	16.67	4.21	33.33	19.23	187.22
Urban	1.43	23.33	9.52	66.67	25.83	201.74
**One-child family**						
Yes	1.33	18.52	8.13	50.00	26.67	198.54
No	1.33	18.18	4.44	41.67	15.15	185.88
**Education of mother**						
Junior high school or below	1.08	22.22	8.00	50.83	26.67	191.50
Senior high school/college	1.39	20.00	7.28	51.73	15.48	191.59
Undergraduate or above	1.33	16.04	4.17	39.35	23.68	190.23
**Education of father**						
Junior high school or below	1.67	21.05	8.33	55.56	14.29	177.78
Senior high school/college	1.39	22.22	6.67	55.56	23.81	219.33
Undergraduate or above	1.08	14.64	4.08	28.33	17.29	171.10
**Average monthly household income**						
<5,000 CNY	1.41	24.40	9.52	51.19	15.48	191.77
≥5,000 CNY and <10,000 CNY	1.43	18.18	6.94	55.56	26.26	199.00
≥10,000 CNY	1.14	14.29	4.17	28.33	15.28	187.04
**Totally**	1.33	18.52	6.67	45.80	20.52	191.59

**Table 4:** Comparison of direct medical cost per day per patient after the GD than before (CNY).

	**Consultation**			**Lab**			**Drugs**			**Hospitalization**			**rehabilitation**			**Totally**		
	**CNY**	**De**	***Z***	**CNY**	**De**	***Z***	**CNY**	**De**	***Z***	**CNY**	**De**	***Z***	**CNY**	**De**	***Z***	**CNY**	**De**	***Z***
Diagnostic age^*^																		
<1 years old	0.69	73.07	−8.297^*^	6.27	81.19	−10.411^*^	4.38	51.80	−5.271	10.22	89.78	−9.21^*^	14.78	−106.98	−1.386	77.14	75.26	−10.222^*^
≥1 years and <3 years	0.65	30.57	−3.698^*^	4.07	66.07	−7.839^*^	2.36	53.21	−3.740^*^	3.60	86.34	−4.959^*^	24.20	27.41	−0.759	66.78	49.30	−4.705^*^
≥3 years	0.28	8.79	−0.972	3.55	61.63	−2.496	1.81	45.77	−1.006	1.24	83.57	−0.831^*^	40.57	−77.70	−2.451	79.37	8.97	−0.693
**Sex**																		
Male	0.47	63.67	−6.532^*^	3.80	80.45	−10.138^*^	2.63	60.51	−5.104^*^	3.91	90.14	−6.538^*^	26.69	12.29	−0.751	72.57	60.29	−7.024^*^
Female	0.69	50.10	−5.972^*^	6.49	62.93	−8.499^*^	4.24	36.33	−3.810^*^	7.27	86.92	−7.757^*^	15.26	−6.85	−0.516	70.38	64.64	−7.655^*^
**County**																		
Suburban	0.63	49.35	−6.436^*^	4.24	74.53	−10.699^*^	1.95	53.70	−4.160^*^	1.60	95.21	−7.797^*^	24.41	−26.94	−0.472	70.20	62.50	−7.832^*^
Urban	0.52	63.64	−6.125^*^	8.13	65.16	−7.910^*^	5.17	45.75	−4.924^*^	10.33	84.50	−6.391^*^	14.43	44.15	−1.237	74.94	62.85	−6.748^*^
**One-child family**																		
Yes	0.46	65.28	−7.624^*^	4.62	75.06	−10.034^*^	3.57	56.08	−4.439^*^	4.24	91.52	−7.254^*^	19.57	26.63	−1.129	71.18	64.15	−7.099^*^
No	0.70	47.49	−4.814^*^	5.25	71.14	−8.334^*^	3.06	31.22	−4.539^*^	5.46	86.89	−7.037^*^	22.59	−49.13	−0.735	75.31	59.48	−7.543^*^
**Education of mother**																		
Junior high school or below	0.40	63.08	−5.105^*^	5.86	73.65	−6.881^*^	4.25	46.86	−3.538^*^	7.70	84.86	−5.165^*^	14.99	43.79	−1.181	65.58	65.75	−5.406^*^
Senior high school/college	0.56	59.71	−5.659^*^	5.02	74.92	−8.569^*^	3.95	45.64	−3.407^*^	6.99	86.48	−6.403^*^	24.41	−57.70	−0.871	85.46	55.40	−6.439^*^
Undergraduate or above	0.73	44.88	−4.676^*^	4.42	72.41	−7.488^*^	1.88	54.81	−4.159^*^	2.14	94.55	−5.862^*^	22.34	5.66	−0.601	62.18	67.31	−6.063^*^
**Education of father**																		
Junior high school or below	0.42	74.64	−4.925^*^	0.98	95.35	−6.485^*^	0.75	90.98	−3.373^*^	8.10	85.42	−4.948^*^	8.90	37.67	−0.261	66.78	61.24	−4.695^*^
Senior high school/college	0.56	59.55	−6.144^*^	0.82	96.29	−8.751^*^	0.50	92.47	−4.77^*^	5.53	90.04	−7.090^*^	20.32	14.68	−0.886	75.16	65.70	−7.573^*^
Undergraduate or above	0.73	32.11	−4.149^*^	0.63	95.72	−7.523^*^	0.40	90.24	−2.740^*^	1.12	96.04	−5.198^*^	21.01	−21.50	−0.554	68.99	59.38	−5.220 ^*^
Average monthly household income^*^																		
<5,000 CNY	0.57	59.34	−4.124^*^	1.06	95.64	−5.960^*^	0.93	90.27	−2.551^*^	12.32	75.94	−4.773^*^	12.42	19.73	−0.554	75.75	60.46	−5.582^*^
≥5,000 and <10,000 CNY	0.42	70.42	−6.960^*^	0.62	96.57	−9.703^*^	0.39	94.37	−5.591^*^	4.15	92.54	−7.465^*^	15.47	41.09	−1.910	64.48	66.54	−8.003^*^
≥10,000 CNY	0.77	32.82	−3.880^*^	0.92	93.58	−7.091^*^	0.42	89.88	−2.880^*^	0.00	100.00	−5.020^*^	25.23	−65.16	−1.669	74.94	58.50	−4.221^*^
**Totally**	0.57	57.41	−8.847^*^	0.78	95.77	−13.233^*^	0.57	91.39	−6.387^*^	4.19	90.85	−10.10^*^	17.95	12.51	−0.417	71.45	62.48	−10.346^*^

*De, Descend (%)*.

* *Showed there was statistical significance between different characteristics after the GD or the cost of one characteristic before and after GD*.

The Kruskal–Wallis H test indicated that the younger the child was at the time of diagnosis, the greater the benefits of genetic diagnosis. If the diagnostic age was younger than 1 year old, the average daily medical expenses would be reduced by 75.26% from 311.79 CNY before GD to 77.14 CNY after GD. If diagnostic age was 1–3 years old, the average daily medical expenses would be reduced by 49.30% from 131.73 CNY before the GD to 66.78 CNY after GD. If diagnostic age was ≥3 years old, there was no statistically significant difference before and after the GD. The impact of other characteristics on cost after diagnosis was not statistically significant, including gender, one-child family, and parents' education level. In addition, the higher monthly household income group reduced the cost by 58.50% per day per patient, which was less than other groups (*P* = 0.000).

## Discussion

In general, NDDs place heavy burden on families. There was no comparability for the total cost because the age composition was inconsistent between our study and other research (4, 5). However, the daily cost per patient revealed important meaning. On the whole, after GD, the average daily total direct medical expenses fell from 191.59 CNY to 71.45 CNY, declining by 62.48%. The descending range of the total cost was higher than in the study by Walsh et al. (22.5%) (11) but lower than in the study by Fogel et al. which showed that the daily cost could reduced by 79.34% after a GD of intellectual disability (8). The reason for the difference could be that GD may harbor an intrinsic “end-of-trajectory” effect, regardless of the diagnosis, downstream medical interventions decrease substantially in both number and costs (5). Of course, this effect was also obvious in China. In addition, our study not only showed the benefits of receiving GD, but also revealed that the younger the age of diagnosis, the greater the benefit. The median daily medical cost fell by 75.26, 49.30, and 8.97% in the age range of <1, 1–3, and ≥3, respectively. Therefore, GD should be promoted in patients with NDDs as soon as possible.

For serious disease, insurance premiums and their rates vary depending on the total household income, the number of people living together, age, and place of residence (12). According to the reports, medical assistance can help a lot with cancer and leukemia. In China, reimbursement rates are over 80% for Shanghai and Beijing, while in Fuzhou and Chongqing, they are 60–70% of the total medical expenditure for cancer. In Japan, for leukemia, many municipalities have set zero copayments for medical expenses for children under the age of 15 (13). Regarding rare diseases, patient access to orphan medicinal products (OMPs) and health care services differ greatly between countries. The number of reimbursed OMPs in the selected countries ranges from nearly all available OMPs in the Netherlands, Germany, and France to zero in Armenia (14). Regarding NDDs, there are few special allowances except for general health insurance. In our research, the reimbursement rate was only 15.91%. In addition, our study showed that the mean monthly income per family was 0.6 (0.4,1.0) CNY, and the mean monthly direct medical expenses of each child amounted to 0.69 (0.33, 15,500) CNY. To some extent, once a family has one child with NDD, the family would be in debt. Marcia Pinto indicated that 54% of families with a child that has a rare disease fail to receive any social benefits. The estimate of coping cost signals that 69% families incur loans, and that 22.5% families have had to sell their household assets to cope with treatment cost (15).

In terms of cost categories, in China, the proportion of consultations is often low, such as 1.12% in our study. Little is known about the impact of genetic counseling on patients with epilepsy and their family members, but in other patients, genetic counseling yields benefits in terms of knowledge, risk perception accuracy, and anxiety reduction (16). In the Netherlands, the proportion of consultations accounts for 14% of the total cost for intellectual disability (5). Comparing this with the research of Vrijenhoek et al. another phenomenon is worth noting: Our study showed that the proportions of hospitalization and rehabilitation are 29.81% and 43.08%, respectively, but 48% and 0%, respectively in the research of Terry Vrijenhoek (5). We concluded that the cost of rehabilitation is included in hospitalization, which could result in reimbursement in the Netherlands. In China, the cost of rehabilitation is mainly undertaken by patients themselves. Therefore, they often undergo rehabilitation in outpatient clinics, which cost less. Even so, the cost of rehabilitation for NDD children is dominant. Therefore, the government should pay more attention to rehabilitation, including medical insurance and the construction of rehabilitation institutions.

Another common phenomenon is the uneven distribution of medical resources. In the United States, nearly 1,400 Health Resources and Services Administration-funded health centers (HCs) serve low-income and underserved populations, and more than 600 of these HCs are located in rural areas (17). Thailand is an example of developing country with limited resources and a low geneticist-to-population ratio with only 17 clinical geneticists in 2017 (18). China is a nation with growing public interest in medical genetic technology. However, the genetic testing and consulting centers are mainly distributed in large cities. Our research showed that each patient with NDDs spends 6 (4,12) visits in outpatient care and 2 (1,4) visits in inpatient care to confirm a GD, and 58.6% patients have to receive testing in another province in order to obtain a GD.

One special aspect of our study is the large cost ranges when taking all cost categories into account. The cost categories included not only direct medical cost but also non-healthcare cost and indirect medical cost, which have not been reported in other economics studies. We found that the non-healthcare cost and indirect medical cost of GD per patient is greater than the direct medical cost (16.79 vs. 12.27 10,000 CNY), which was similar to the research of Pinto et al. where the loss of earnings exceeded 100% for rare diseases (15). The burden of care placed on parents was significant in meeting these complex challenges (20). Parents must become experts with care providers in addressing pervasive health and social needs (19). Many parents, especially mothers, need to devote time to their children, which leads to a loss of work. In the research of Genevieve, among 15 parents of patients with rare NDDs, 13.33% of parents stayed home full time with their kids (19).

This study is the first to illustrate the impact of NDD on families in China. The daily direct medical cost after GD was 62.48% lower than before. Hence, the government should encourage clinicians to make GD early. There are also some limitations of this study. First, there were no undiagnosed group controls for which we were still in the process of gathering information. Second, we only took economic impact into consideration, ignoring the mental stress of the family. We will examine these matters in future research.

In general, NDDs cause heavy pressure regarding the burden of disease for parents with NDD patients. We call for the first diagnosis to be made early and to reduce direct medical expenses. Secondly, medical insurance assistance should be increased to reduce the burden of disease for families. Thirdly, social support should be increased for families with NDD patients.

## Data Availability Statement

The original contributions presented in the study are included in the article/supplementary materials, further inquiries can be directed to the corresponding authors.

## Ethics Statement

The studies involving human participants were reviewed and approved by the Ethics Committee of Maternal and Child Care Hospital of Hunan Province. Written informed consent to participate in this study was provided by the participants' legal guardian/next of kin.

## Author Contributions

DX and RD designed the research, analyzed the data, and wrote the article. CL recruited patients and helped carry out the survey. ZX, AW, LX, JF, JuW, YZ, HY, JiaW, and HX collected and checked the data. XM, HW, and JinW helped with the design and control for the quality of the research. All authors reviewed and approved the article.

## Funding

This work were supported by the Major Scientific and Technological Projects for collaborative prevention and control of birth defects in Hunan Province (2019SK1011, 2019SK1014, and 2019SK1010), the Foundation of the Ministry of Health of Hunan Province, China (202212034013), and the National Key Research and Development Program of China (No. 2016YFC1306201).

## Conflict of Interest

The authors declare that the research was conducted in the absence of any commercial or financial relationships that could be construed as a potential conflict of interest.

## Publisher's Note

All claims expressed in this article are solely those of the authors and do not necessarily represent those of their affiliated organizations, or those of the publisher, the editors and the reviewers. Any product that may be evaluated in this article, or claim that may be made by its manufacturer, is not guaranteed or endorsed by the publisher.
